# Frequency of dilaceration in a mexican school-based population

**DOI:** 10.4317/jced.54368

**Published:** 2018-07-01

**Authors:** Constantino Ledesma-Montes, Juan-Carlos Hernández-Guerrero, María-Dolores Jiménez-Farfán

**Affiliations:** 1 Clinical Oral Pathology Laboratory. Facultad de Odontología. Universidad Nacional Autónoma de México. México City 04510. Mexico; 2 Laboratory of Immunology. Facultad de Odontología. Universidad Nacional Autónoma de México. México City 04510. Mexico

## Abstract

**Background:**

The aim of this study was to record the frequency of dilaceration in patients attending our institution and to analyze the possible associated factors.

**Material and Methods:**

Orthopantomograms from all patients attended in our institution were reviewed and those cases of dilaceration were selected. Documented data were age, gender, diagnosis, location and involved teeth. Data on possible etiological factors was also recorded.

**Results:**

125 dilacerated teeth in 99 patients were found. Dilacerations were more commonly detected in females and in maxillary teeth. Maxillary 2nd bicuspids and lateral incisors were the more commonly affected teeth and were more common in teeth with predecessors (anterior teeth and bicuspids). Traumatic episodes and caries of the predecessor teeth was mentioned but never were related with affected teeth.

**Conclusions:**

Data from the studied population are different compared to previously published studies. Our results support the point of view that the occurrence of dilacerated teeth could be related to limited availability of space to allocate the erupting teeth in the dental arch and perhaps to the possibility of the tooth to rotate, preventing eruption.

** Key words:**Developmental alterations, dental developmental alterations, root dilaceration.

## Introduction

Dilaceration is a rarely observed developmental alteration of the dental root first described by Tomes (1). The Glossary of Terms of the American Academy of Endodontists defines dilaceration as “a deformity characterized by displacement of the root from its normal alignment with the crown; may be a consequence of injury during tooth development. Common usage has extended the term to include sharply angular of deformed roots” (2). Two theories try to explain its origin: The first is trauma to the primary tooth resulting in tooth germ displacement in such a way that the permanent tooth root will develop an angle (3). When a traumatic factor is not known, a second theory deals with a developmental disturbance of unknown origin (4).

There are some studies on the frequency of dilaceration in several populations (4-11). According to these publications, this developmental alteration is more frequently found in posterior mandibular areas (5-8), comprises from 0.32% to 16% of the studied populations (9,10) and it has no gender preference (7-9). According to Jafarzadeh and Abbot (12) several associated factors, could be implicated in development of this condition.

The aim of this study was to record and analyze the frequency of dilaceration in patients attending our institution and analyze the possible factors associated to its development.

## Material and Methods

This study included all the patients who sought stomatological attention during one year in the Admission and Diagnosis Clinic, Facultad de Odontología, UNAM. Ethics Committee analyzed and approved the protocol and all patients and parents signed a Letter of Consent giving permission to use data for research purposes. At first appointment, all the patients received an oral and maxillofacial examination. This assessment included careful observation and palpation of the soft and hard oral tissues and careful review of the head and neck area. A panoramic radiograph was made to all patients and all radiographs were reviewed. A tooth was considered as having a dilaceration towards the mesial or distal direction if there was a 90° angle or greater along the axis of the tooth or root (5). Orofacial direction of the dilacerations was determined by evaluating the bull’s eye appearance of the root, which results from the root deviation of 90° or more in a buccal-lingual/palatal direction. A dilacerated multirooted teeth was recognized when at least one dilacerated root was detected and it was counted as one case and to diagnose dilaceration the affected tooth must be fully developed. All readings were made independently by two Oral Pathologists with more than 30 years’ experience (CL-M & JCH-G) Before starting the investigation, intra-examiners calibration was done by reading 100 radiographs including cases of dilacerated teeth. Two weeks and one month after the first calibration, both examiners read an extra-sample of 100 panoramic radiographs containing dilacerations and a 100% agreement was obtained. Documented data were age, gender, diagnosis, location and involved teeth. All the possible etiological factors that could be implicated in development of this condition mentioned by Jafarzadeh and Abbot (12) were included and analyzed. All findings were recorded in specially designed forms. Data on third molars were not included in the study. Student T test was applied and *p*<0.05 was considered of statistical significance.

## Results

From the 6,340 patients attending our clinic, we selected those cases with teeth showing dilacerated roots. They were 125 teeth in 99 patients (1.6%). Of them, 68 were females (68.7%) and 31 were males (31.3%). Difference between genders was statistically significant (*p*<0.05). Patient ages were between 7 and 80 years (mean age= 39 years). This developmental alteration was more frequently seen in patients between 11 and 30 years age (40.4%). Statistical significance was found comparing data between this group and older patients (*p*<0.05). It was observed that frequency of dilacerated teeth decreased in patients aged 51 years and older. Frequency of patients’ gender and age is shown in  Table 1 and data on involved teeth is in table 2. 123 teeth were of the permanent formula (98.4%) and 2 were deciduous teeth (1.6%). As it is in  Table 2, This malformation appeared more frequently in maxillary teeth (n= 68; 54.4%) compared with mandibular cases (n=57; 45.6%), but statistical significance was not found (*p*>0.05). Bicuspids were more frequently affected, followed by molars and anterior teeth. Comparing all these figures statistical significance was obtained (*p*<0.05). The most frequently affected teeth were maxillary 2nd bicuspids and maxillary lateral incisors (n= 17; 13.6% respectively), followed by maxillary 1st bicuspids and mandibular 2nd molars (n= 15; 12% respectively). According to the recovered data, dilaceration was more common in teeth with deciduous predecessors (anterior teeth and bicuspids; 68%), compared with frequency of this developmental alteration recorded in other teeth. Statistical significance was found (*p*=0.005) comparing data from both groups. From the etiological factors mentioned by Jafarzadeh and Abbot (12), the patients mentioned trauma and caries in deciduous teeth but none of them occurred in the area of the dilacerated tooth.

**Table 1:**
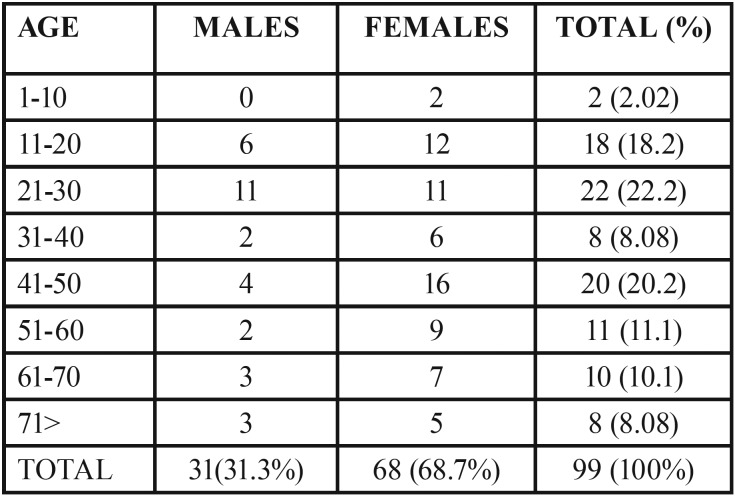
Age and gender of the studied patients.

**Table 2:**
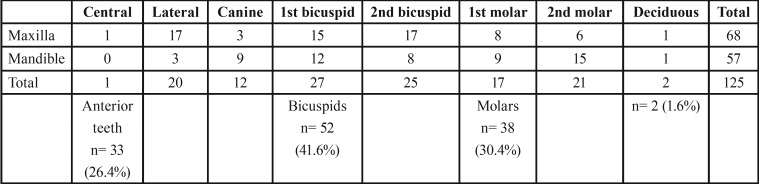
Frequency of dilacerated teeth in the studied population.

## Discussion

Dilaceration is a well-known developmental alteration consisting in a change of the normal alignment of the dental root and crown. This developmental alteration has not been extensively studied and data on its origin, frequency, gender preference, most frequently affected group of teeth and most commonly involved teeth is controversial (3-11).

It was reported that dilaceration was more commonly detected in females but to date, frequency on gender has not been elucidated. In this study, our data showed female preference agreeing with other reports (6) and disagreeing with previous studies informing that dilaceration had no gender predilection (7-9) or indicating male preference (5). Additionally, no agreement on preference of location exists. According with previous reports (6,8) data from this study showed it was more frequent in maxilla. This finding contrasts with Colak et al study, who found it was more frequent in mandible (9) and with results from other studies reporting equal maxillo-mandibular frequency (5,7). Also, data on frequency by individual tooth is controversial among the previously reported data and also there are published differences in frequency considering the group of more frequently affected teeth (5-9).

Interestingly, in this study dilacerations were more common in teeth with deciduous predecessors (bicuspids and anterior teeth) compared with frequency in other teeth with no precursors. This finding suggests that development of dilaceration could be associated to reduced availability of space in the dental arch to allocate the erupting teeth, and perhaps to the possibility of the tooth to rotate preventing adequate eruption. This theory is supported by our findings and those from the Gupta *et al.* study (11) showing that more dilacerations were detected in the bicuspid and incisor areas. This knowledge opens a new perspective to the medical and dental professional during radiographic review of the patients, being able to suspect if a patient presents lack of space and refer the patient to a specialist.

## Conclusions

1. Results from this study support the concept that origin of this developmental alteration is associated to reduction in the availability of space in the dental arch to allocate the erupting teeth.

2. Knowledge on the frequency of dilaceration will benefit preventing the problems associated to endodontic, orthodontic and surgical treatments.

3. Data from this study shows that frequency of this developmental alteration is different compared with studies from other populations.

## References

[1] Tomes J A course of lectures on dental physiology and surgery (lectures I-XV).

[2] Glossary of Endodontic Terms.

[3] van Goo AV (1973). Injury to the permanent tooth germ after trauma to the deciduous predecessor. Oral Surg Oral Med Oral Patol.

[4] Andreasen JO, Sundström B, Ravn JJ (1971). The effect of traumatic injuries to primary teeth on their permanent successors. I. A clinical and histologic study of 117 injured permanent teeth. Eur J Oral Sci.

[5] Hamasha AA, Al Khateeb T, Darwazeh A (2002). Prevalence of dilaceration in Jordanian adults. Int Endod J.

[6] Udoye CI, Jafarzadeh H (2009). Dilaceration among Nigerians: prevalence, distribution, and its relationship with trauma. Dent Traumatol.

[7] Miloglu O, Cakici F, Caglayan F, Yilmaz AB, Demirkaya F (2010). The prevalence of root dilacerations in a Turkish population. Med Oral Patol Oral Cir Bucal.

[8] Bodrumlu E, Gunduz K, Avsever H, Cicek E (2013). A retrospective study of the prevalence and characteristics of root dilaceration in a sample of the Turkish population. Oral Radiol.

[9] Malčić A, Jukić S, Brzović V, Miletić I, Pelivan I, Anić I (2006). Prevalence of root dilaceration in adult patients in Croatia. Oral Surg Oral Med Oral Pathol Oral Radiol Endod.

[10] Çolak H, Bayraktar Y, Hamidi MM, Tan E, Çolak T (2012). Prevalence of root dilacerations in Central Anatolian Turkish dental patients. West Indian Med J.

[11] Gupta SK, Saxena P, Jain S, Jain D (2011). Prevalence and distribution of selected developmental dental anomalies in an Indian population. J Oral Sci.

[12] Jafarzadeh H, Abbott PV (2007). Dilaceration: review of an endodontic challenge. J Endod.

